# Supplementary material for: Vitamin D Modified DSS-Induced Colitis in Mice via STING Signaling Pathway
Source: Biology (Basel). 2025 Jun 18;14(6):715. doi: 10.3390/biology14060715 (PMC12190092; doi:10.3390/biology14060715)
Supplement: Supplementary file 1 [file biology-14-00715-s001.zip › Supplementary Materials S1: 16s-report of gut microbiota/Groups/diff_analysis/UnivarTestCAZY/figure/barplot/top10.pdf]

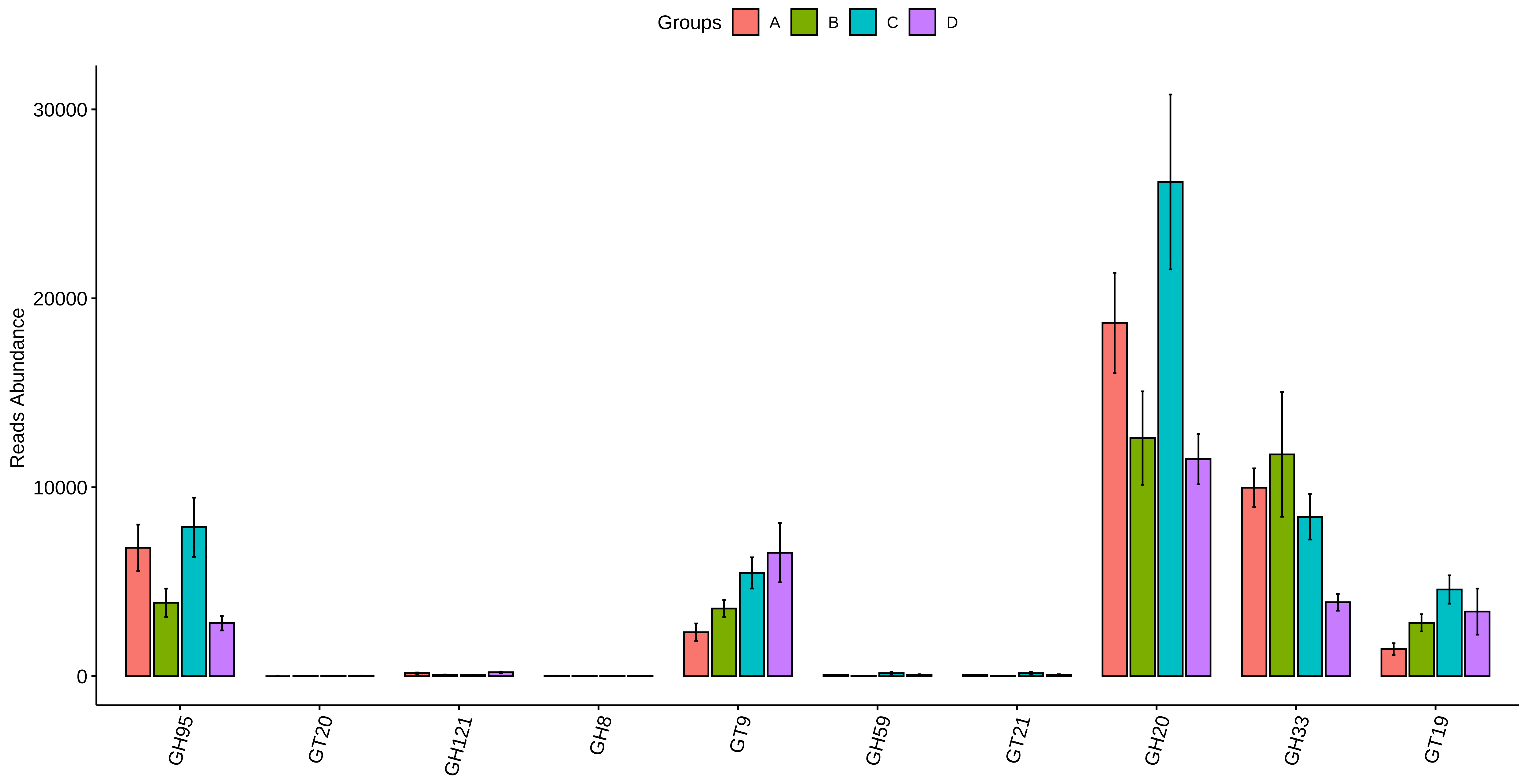
Groups
A
B
C
D
Reads Abundance
30000
20000
10000
0
GH95
GT20
GH121
GH8
GT9
GH59
GT21
GH20
GH33
GT19
